# Large-Field-of-View Visualization Utilizing Multiple Miniaturized Cameras for Laparoscopic Surgery

**DOI:** 10.3390/mi9090431

**Published:** 2018-08-25

**Authors:** Jae-Jun Kim, Alex Watras, Hewei Liu, Zhanpeng Zeng, Jacob A. Greenberg, Charles P. Heise, Yu Hen Hu, Hongrui Jiang

**Affiliations:** 1 Department of Electrical and Computer Engineering, University of Wisconsin-Madison, Madison, WI 53706, USA; jkim724@wisc.edu (J.-J.K.); watras@wisc.edu (A.W.); hliu265@wisc.edu (H.L.); zzeng38@wisc.edu (Z.Z.); yhhu@wisc.edu (Y.H.H.); 2 Department of Surgery, University of Wisconsin School of Medicine and Public Health, Madison, WI 53792, USA; greenbergj@surgery.wisc.edu (J.A.G.); heise@surgery.wisc.edu (C.P.H.)

**Keywords:** large field of view, miniaturized cameras, laparoscopy, bean drop task, surgical skills, video stitching

## Abstract

The quality and the extent of intra-abdominal visualization are critical to a laparoscopic procedure. Currently, a single laparoscope is inserted into one of the laparoscopic ports to provide intra-abdominal visualization. The extent of this field of view (FoV) is rather restricted and may limit efficiency and the range of operations. Here we report a trocar-camera assembly (TCA) that promises a large FoV, and improved efficiency and range of operations. A video stitching program processes video data from multiple miniature cameras and combines these videos in real-time. This stitched video is then displayed on an operating monitor with a much larger FoV than that of a single camera. In addition, we successfully performed a standard and a modified bean drop task, without any distortion, in a simulator box by using the TCA and taking advantage of its FoV which is larger than that of the current laparoscopic cameras. We successfully demonstrated its improved efficiency and range of operations. The TCA frees up a surgical port and potentially eliminates the need of physical maneuvering of the laparoscopic camera, operated by an assistant.

## 1. Introduction

Laparoscopic surgery has gained tremendous popularity over the past decades due to numerous clinical benefits for patients including decreased postoperative pain, decreased wound morbidity, earlier recovery and return to normal activities, and improved cosmesis [1,2,3,4,5]. These clinical benefits are the results of limiting incision size and thus surgical trauma. However, the tradeoffs are the needs for an excellent intracavitary visualization along with a surgeon skill set capable of performing these—often more technically challenging—procedures. Therefore, the ability for carrying out a successful laparoscopic procedure is strongly dependent on the quality and the extent of the intra-abdominal visualization, which is critical for the identification of vital structures, manipulation of tissue, and the surgical performance and for the safe completion of each operation. In the current system, a single laparoscope is inserted into one of the laparoscopic ports to provide this intra-abdominal visualization. However, this paradigm has many well-known drawbacks, in terms of visualization and efficiency of operation.

One drawback in terms of visualization is the limited visual field of a single laparoscope, which severely limits the ability of a surgeon to view the operative field and perceive the surroundings. This increases the possibility of surgical accidents. A panoramic view was suggested to overcome this limitation of the current laparoscope [6,7,8]. Naya et al. showed that panoramic views during laparoscopic surgery shorten the operating time and increase safety [7]. In addition, several attempts have been made to achieve large field-of-view (FoV) imaging systems for biomedical and surgical applications, such as the usage of prisms [9,10], panomorph lenses [11], or mirror attachments [12,13]. These methods, however, suffer from a multitude of issues ranging from aberrations to blind zones. Additionally, they have a common underlying requirement of proximity to the surgical field, which makes them prone to splatter and interference or occlusion from the surgical instruments. The most recent development of imaging devices could potentially improve the FoV and performances of the surgical vision systems. For example, it has been proven that attaching specially designed compound lenses to the front end of an endoscope results in a large FoV and zoom capability [14,15]. Optical beam scanning methods are also promising to increase the FoV of the endoscopic imaging systems [16,17]. Kagawa et al. showed the possibility to increase the FoV of endoscopes by using a compact compound-eye camera [18]. Although these approaches offer advantages in terms of image resolution and aberrations, complicated optical and electronic-mechanical designs of the components lessen their durability and stability. More importantly, they still require coordinated operations of the imaging systems and the surgical tools, through multiple operative ports. Multi-view vision systems have also been developed to improve the quality of surgical visualization. Tamadazte et al. increased the FoV by combining two miniaturized camera with a conventional endoscope [19]. This more developed visual system shortened the procedure time and reduced the number of commands used to operate the robot endoscope holder. Multiple-cameras were used to provide multiple points of view and maintain a three-dimensional perception [20,21]. The proposed multi-view visual systems, however, can only increase the point of view in one direction due to the limitation in camera placement. These can only spread out multiple images and are not able to combine them into a single large one, which degrades the surgical performance, as a result.

Another drawback in terms of efficiency of operations, is that the current laparoscopes must occupy one of the few available surgical ports, exclusively for viewing, and thus prevents further port use for other instruments. As a result, there is always a need to increase the number of ports by one count. Most of the previous visualization systems that have been able to improve visual quality also need to occupy one of the surgical ports.

**Figure 1:**
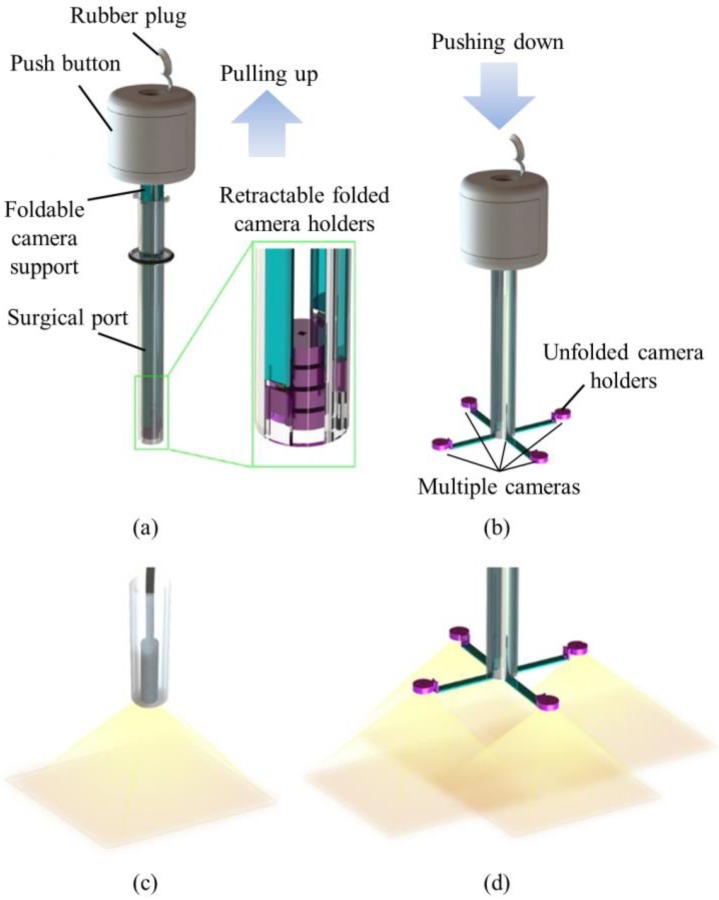
Design and concept of a trocar-camera assembly (TCA). The TCA consists of a push button, foldable camera supports, a surgical port, and miniaturized cameras. (**a**) The miniaturized cameras are stacked within the surgical port when the port is inserted into or extracted from a simulator box. (**b**) Miniaturized cameras are deployed after the surgical port is inserted into the simulator box. Surgical instruments can be inserted through the port because the port is not occupied by the cameras during operation. (**c**) Small field-of-view (FoV) of the current laparoscopic camera. (**d**) Large FoV provided by the multiple cameras.

In order to achieve large FoV and improve the efficiency of operations, we developed a trocar-camera assembly (TCA) and real-time video stitching algorithm. The TCA uses commercial off-the-shelf cameras and a simple and stable mechanical system (see Figure 1a,b). The miniaturized cameras are easily deployed and retrieved using the mechanical system of the TCA. Such convenient deployment and retrieval of the miniature cameras offered by this trocar design is an advantage over our previous attempts to apply multiple cameras at laparoscopic ports [22,23,24]. The TCA can provide a larger FoV compared to a current laparoscopic imaging system that uses a single camera, with the use of multiple miniaturized cameras. (see Figure 1c,d). Unlike the current laparoscopic system, surgical instruments can now be inserted through any surgical port, during an operation, as the port is not exclusively occupied by the laparoscopic camera anymore. The algorithm is able to stitch the videos from multiple cameras without human-detectable latency, and provides smooth, high-resolution videos with a significantly improved FoV. The FoV of the TCA was measured and compared with commercial laparoscopic cameras. To demonstrate the advantages of a large FoV visualization, we implemented a standard bean drop task and a modified one that requires complicated operations in all directions.

## 2. Materials and Methods

### 2.1. Trocar-Camera Assembly (TCA)

The TCA consisted of a push button, foldable camera supports, a surgical port, and miniaturized multiple cameras. We designed the TCA structure. Most of the mechanical parts of the TCA were manufactured by 3D printing and computer numerical control (CNC) machining (Xometry, Gaithersburg, MD, USA), and the torsion springs were made by Michigan Steel Spring Company (Detroit, MI, USA), according to our design. Five Raspberry Pi cameras with flexible cables (Raspberry Pi MINI camera module (FD5647-500WPX-V2.0), RoarKit) were integrated with four foldable camera supports. An extra camera was integrated onto one of the four arms to provide a central, main view of the surgical field (see Results section).

#### 2.1.1. Push Button

**Figure 2:**
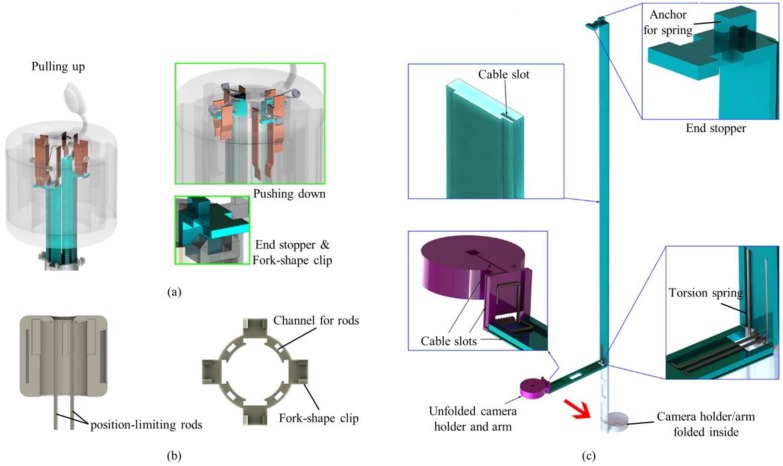
Mechanical system of a TCA. (**a**) The working mechanism of the push button. Pliant plates and torsion springs push down aluminum arms and deploy multiple cameras sequentially. The four fork-shape clips and the end stopper of the arms are utilized to stop and hold the arms. The fork on the clip can separate the pliant plate pairs to bypass the stopper of the arms. (**b**) Cross-section of the push button and top view of the surgical port. (**c**) A foldable camera support consists of two aluminum arms, torsion springs, and a camera holder.

The push button consisted of a top lid, position-limiting rods, pliant plates and torsion springs (see Figure 2a,b). Four position-limiting rods were sintered onto the top lid. The rods were inserted and could slide into the corresponding channels in the surgical port, keeping the push button in position during the deployment and the retraction processes. Sequential deployment and retraction of the cameras were achieved by four pairs of pliant plates and torsion springs of different dimensions, which were also sintered on the top lid. When the push button was pushed down, a longer pair of pliant plates pushed the camera earlier than the shorter plates did. When the first camera unit was in position (stopped by the clip on the surgical port), the pliant plate pair was separated by the fork structure of the clip and bypassed the camera support, and its torsion spring got bent while the shorter sheets continuously pushed the other cameras. In this manner, the cameras could be deployed out of the port one by one. Retraction of the cameras was performed in a reversed procedure: shorter spring pulled the camera back earlier than the longer springs did.

#### 2.1.2. Surgical Port

The surgical port of the multi-camera laparoscope had a 150-mm long plastic tube with a 26 mm outer diameter. Our design allowed for embedded camera supports, so the surgical port contained four trenches on the inner surface of the arms and four channels inside the sidewall of the port (see Figure 2b). On the upper end of the port, four fork-shape clips were utilized to stop and hold the arms, and the fork on the clip could separate the pliant plate pairs to bypass the stopper of the arms.

#### 2.1.3. Foldable Camera Support

The foldable camera support consisted of two arms, a camera holder, and right-angled torsion springs (see Figure 2c). Each support could deploy one camera unit, and in this design example, four supports were utilized. Right-angled torsion springs connected the components with each other. When it was retracted inside the port, the channel forced the springs straight down so that cameras could be stacked up in the tube. Once the cameras and short arms were pushed out of the surgical port, torsion springs restored the right angle and unfolded the cameras as shown in Figure 1.

Each long arm had an end stopper that could be locked by the clip on the surgical port. The spring anchor on top of the stopper could be hooked on one end of the torsion spring in the push button. The rear surface (the surface touching the port) of the other end had a groove for mounting the right-angled spring.

### 2.2. Demonstration Setup

**Figure 3:**
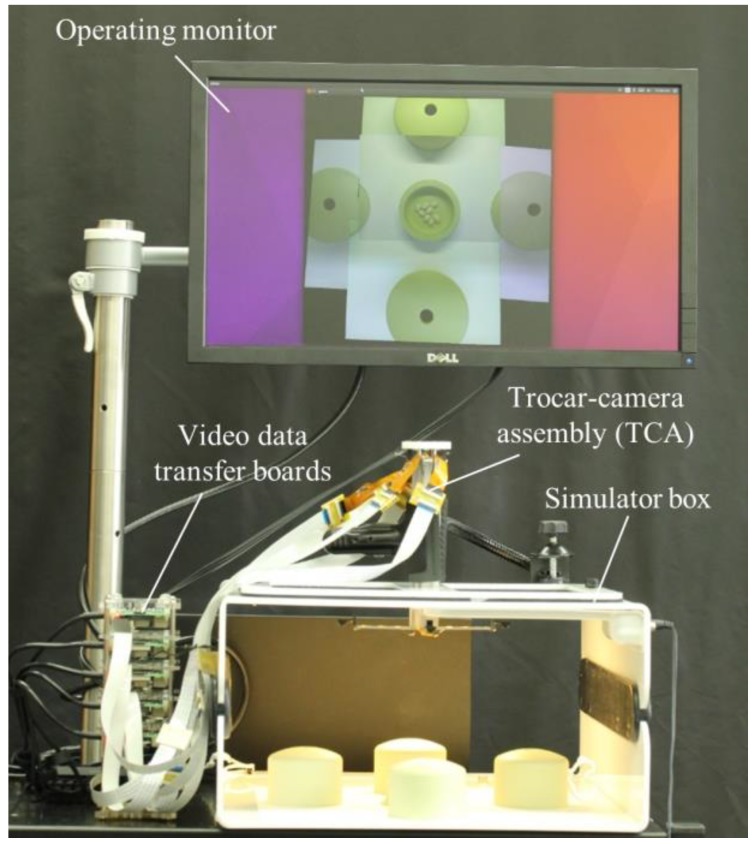
Demonstration setup. The TCA is inserted into the simulator box. Video data from five cameras are transferred to a computer through video data transfer boards and then the stitched video is shown on the operating monitor.

The demonstration setup consisted of a TCA, a simulator box, an operating monitor, video data transfer boards, and a computer running the video stitching program (see Figure 3). The TCA was inserted through a simulator box and five cameras were deployed by the mechanical system of the TCA. Five Raspberry Pi 3 model B boards (Adafruit, New York, NY, USA) were used for video data transfer. Flex cables (Flex Cable for Raspberry Pi Camera or Display—300 mm, Adafruit) and ZIF connectors (15 pin to 15 pin ZIF 1.0 mm pitch FFC cable extension connector, Rzconne) were used to connect the miniaturized cameras and the Raspberry Pi boards. The computer and Raspberry Pi boards were connected by Ethernet cables and an Ethernet switch (JGS524, Netgear, San Jose, CA, USA). The operating monitor displayed real-time stitched video after the computer completed the video stitching process.

### 2.3. Image Processing for Video Stitching

**Figure 4:**
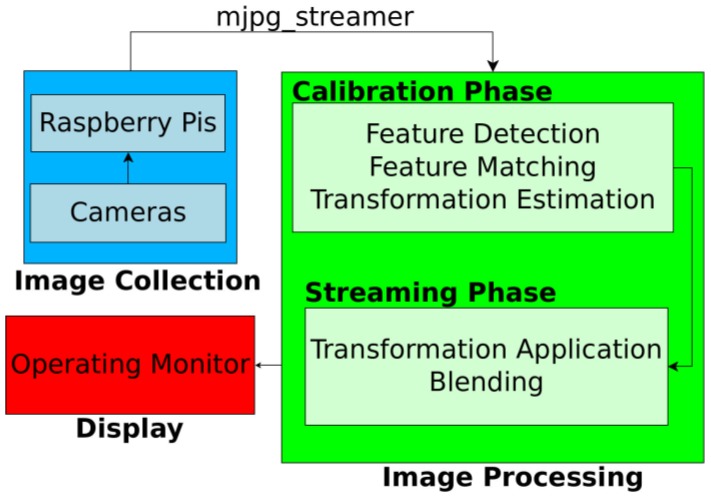
Flowchart of the real-time video stitching process.

Figure 4 shows the flowchart of the real-time video stitching process. First, we collected images from the five Raspberry Pi cameras, and the five Raspberry Pi boards streamed these videos by using an MJPG-streamer. The resolution and frame rates of each streamed video were 640 × 480 pixels and 30 frames per second (fps), respectively. The video data from the boards were transferred to the computer running the stitching program, through Ethernet cables. The stitching program combined five videos in real time to show a large FoV mosaic. The detailed process of stitching program is described in the following section. The operator watched the real-time stitched video on the operating monitor during the task.

The general process of video stitching follows these steps. First, we find distinguishable landmarks in a set of incoming video frames by using the speeded up robust features (SURF) algorithm [25], which can identify recognizable regions in an image. This allows us to identify those features in other images. Then a feature matching algorithm must attempt to find features that show up simultaneously in images from multiple cameras. Once we have identified a sufficient number of feature matches, we attempt to find a transformation that can be applied to one image such that the features detected in that image are moved to their corresponding coordinates, as seen from another camera. This transformation is subsequently applied to the full source image so that both images now represent the same projection from the scene to the camera. Finally, the images are blended together to form a mosaic.

Initial stitching methods based on the work by Szeliski [26] proved to be too slow for real-time video work, as the amount of time required to detect feature points alone introduced significant latency. As a remedy, we instead split the task of image stitching into an initialization phase and a streaming phase as proposed by Zheng [27].

The initialization phase, which takes several seconds (e.g., 5–6 s), served to allow us to perform as much of our computations as possible ahead of time to remove computation while the video was streaming. During this phase, we computed feature points from the scene and used them to generate pixel correspondences between the cameras. We chose a single camera to be our main view and then used the detected feature points to determine which transformations could be used to best align the images. The chosen transformations were stored for the streaming phase.

The streaming phase then only required that the chosen transformations be applied to each incoming video frame, as it was received, and that the images be blended together. There is a variety of blending algorithms aimed at hiding image seams for more realistic-seeming views. However, we chose to use the most basic blending method in order to ensure fast computation and clarity of our recorded scene. With our method, we chose an ordering of the cameras, then each camera was placed in order, onto a unified canvas. In this way, no extra artifacts were introduced by the blending algorithm.

In order to minimize the latency and speed up the stitching process, we used a multi-thread method. Eleven threads were created in this study. Five threads were used to capture images from five cameras. Each one of these five threads was used to transfer the data from one camera and skip the frames that could not be processed in time, so the image being used for stitching were always up to the latest frame. This eliminated latency as much as possible. Four threads were used to compute the homographies and warp the images. Each one of these four threads was used to warp images from a side-view camera to the perspective of the main-view camera, so that the main stitching process could be executed in parallel. This significantly reduced the overall time required for each frame. One thread was used to transfer images between different threads and display the final stitched panorama. The last thread was used to record the camera streams, the panorama stream and the output as video files.

### 2.4. FoV Measurement

To compare the obtained FoV from the TCA and from current laparoscopic cameras (5 mm and 10 mm 0° laparoscopic cameras), we captured images of grid lines that had 25 mm periodic lines with numbers. Both cameras were placed 165 mm away from the grid lines.

### 2.5. Standard and Modified Bean Drop Task

A simulator box (Fundamentals of Laparoscopic Surgery (FLS)) was used to implement a standard and modified bean drop task. The saucer, inverted cups, and beans used in these tasks were commercial products (SIMULAB, Seattle, WA, USA). We used a 5 mm atraumatic grasper (Ethicon, Somerville, NJ, USA) to grasp the beans.

A standard bean drop task is one of the basic skill tasks for laparoscopic training. The standard bean drop task requires an operator to grasp the beans by a grasper, move the beans 15 cm from the saucer to the inverted cup and drop them into the hole of the inverted cup (see Results section).

We also developed a modified bean drop task which consisted of one saucer with beans and four inverted cups placed in four different positions (see Results section). The goal of this task was for an operator to grasp and move each bean 10.5 cm from the saucer to the inverted cups, and then drop them into the holes of the inverted cups. Therefore, this modified bean drop task can evaluate the operating skill in all directions.

## 3. Results

**Figure 5:**
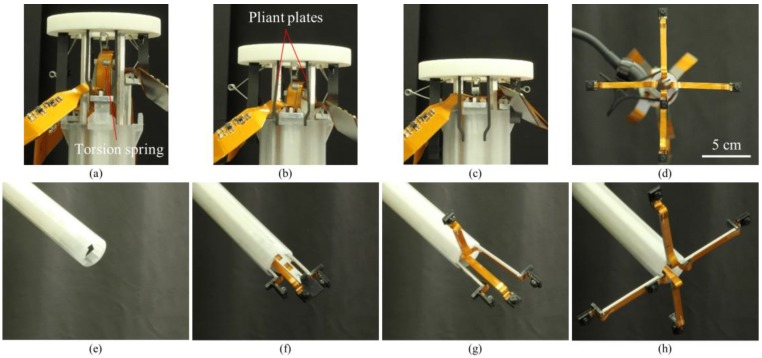
Deployment and retraction of the multiple cameras. (**a**–**c**) Mechanical system of the push button. Stacked cameras can be deployed and retrieved in a single action within several seconds. (**d**) Camera positions after deployment. (**e**–**h**) The mechanical system of the foldable camera supports. Four foldable camera supports and five cameras are sequentially deployed and retrieved. The inner side of the port is not occupied by the cameras during operation and allows insertion of surgical instruments.

The four aluminum arms and five cameras were easily, and sequentially, deployed and retrieved by the mechanical system of the TCA within several seconds, as shown in Figure 5. Thus, the inner side of the port was not occupied by the cameras during the operation and could still be utilized by the instruments. Therefore, our system could reduce the number of ports or free up a surgical port, which would be a prominent advantage. Moreover, this stacked camera arrangement could efficiently use the small inner space of the laparoscopic port and maximize the camera size.

Our video stitching program used the video data from five individual cameras and combined these five videos in real time to show a large FoV mosaic. A projective transformation was calculated using matched feature points for mapping each image from different cameras into a single coordinate system. One of the five cameras placed at the center part of the camera array was considered to be the main view and then the required transformations were computed to map the images from five different cameras onto the coordinate system of the main view. As our cameras remained stationary, relative to each other and the scene plane, during the video streaming we simply applied each of the calculated transformations and merged the images together. Our stitching algorithm was able to provide videos at approximately 26 fps, comparable to the 30 fps offered by the individual cameras. The total latency of the stitching process was approximately 200 ms and surgeons noted that they did not notice lag from their actions to the display (see Figure 8, Video S1).

**Figure 6:**
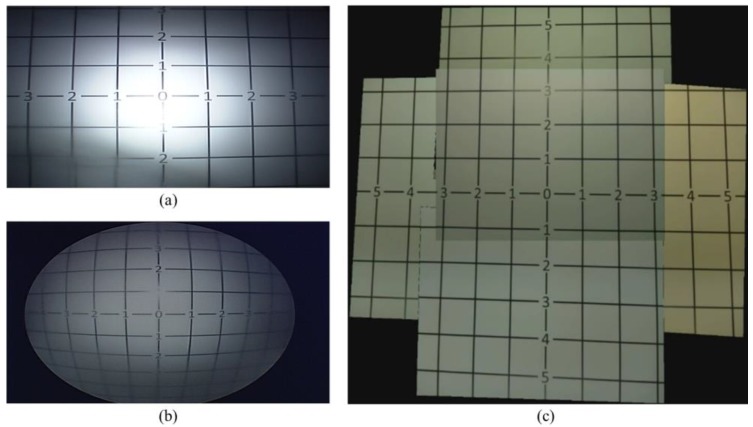
FoV measurement using grid lines printed on a paper sheet. Captured images from (**a**) a 10 mm 0° laparoscopic camera, (**b**) a 5 mm 0° laparoscopic camera, and (**c**) the TCA. The TCA can cover larger FoV compared with commercial laparoscopic cameras. Note the distortion in the view acquired from the 10 mm 0° laparoscopic camera (a) and the 5 mm 0° laparoscopic camera (b).

We compared the FoV of commercial laparoscopic cameras with that of the TCA by using grid lines (see Figure 6). Figure 6a–c shows captured images from a 10 mm 0° laparoscopic camera, a 5 mm 0° laparoscopic camera, and the TCA, respectively. As shown in Figure 6, the TCA covers a larger area and detects more grid lines than current laparoscopic cameras. The 10 mm and 5 mm 0° laparoscopic cameras can detect up to number 3 and 4 grid lines, respectively. Compared with current laparoscopic cameras, the TCA can detect up to number 5 grid lines without image distortion. Based on this measurement, the FoV of the 10 mm, 5 mm 0° laparoscopic cameras, and the TCA is 49°, 62°, and 74°, respectively. The FoV of the TCA increased by 51% and 19%, compared with 10 mm and 5 mm 0° laparoscopic cameras, respectively. We also can compare visible imaging area using the number of visible squares. The number of visible squares of the 10 mm, 5 mm 0° laparoscopic cameras, and the TCA was 36, 56, and 84, respectively. The visible area of the TCA increased by 133% and 50%, compared with 10 mm and 5 mm 0° laparoscopic cameras, respectively.

**Figure 7:**
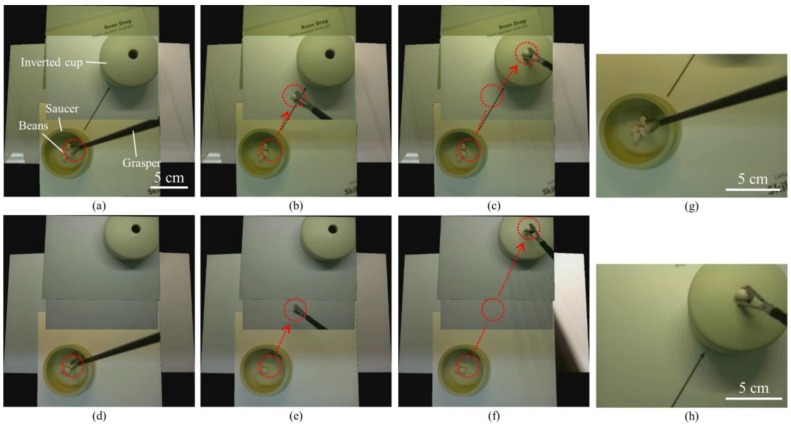
Captured images from a real-time stitched video during a standard bean drop task in the simulator box by using the TCA (**a**–**c**), and a standard bean drop task with longer moving distance and without an arrow mark (**d**–**f**). We successfully performed a standard bean drop task by using the real-time stitched video without any physical camera maneuver. The red dotted circles and arrows in each figure show initial and end positions, and the trajectories of the moving beans grasped by a grasper. (**g**,**h**) The images from two single cameras show that a single camera cannot cover the saucer and the inverted cup simultaneously.

We successfully performed two different sets of a standard bean drop tasks in a simulator box by using the TCA (see Figure 7, Video S2, Video S3). One set of the standard bean drop task was performed with an arrow mark guiding the way from the saucer to the inverted cup (Figure 7a–c); the other task was performed without an arrow mark and with longer moving-distances (Figure 7d–f). In both tasks, the operator could track the whole motion of beans and visualize the beans and the inverted cup simultaneously through the TCA. Unlike the current laparoscopic system, the TCA does not need the arrow mark for guiding. The bean was grasped by a grasper (Figure 7a,d) and dropped into the hole of the inverted cup (Figure 7c,f). The beans and the inverted cup are 155 mm and 115 mm away from the camera array, respectively. The red dotted circles indicate the start and end positions and the red arrows show the trajectories of the moving beans. During the task period, the operator continuously watched the real-time stitched video on the operating monitor and used only one hand to perform the two sets of the standard bean drop tasks, without any physical camera maneuver. This is a clear advantage over current laparoscopic cameras, where there is a need for separate camera operation by an assistant. In contrast, the captured images from two single cameras (Figure 7g,h) show that a single camera cannot cover the saucer and the inverted cup concurrently.

**Figure 8:**
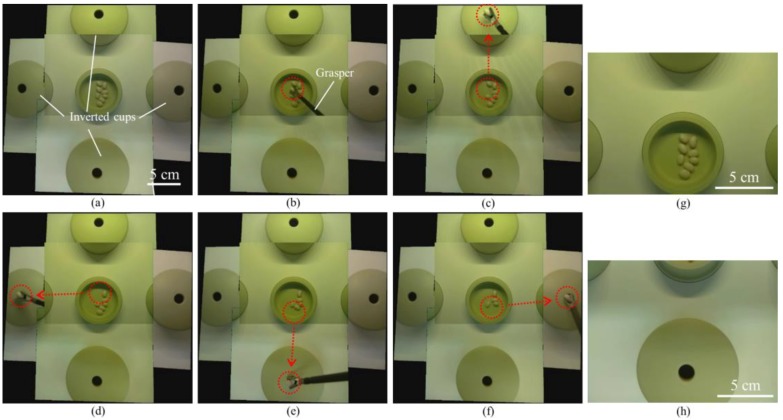
Captured images from a real-time stitched video during a modified bean drop task in the simulator box by using the TCA. (**a**) Setup of a modified bean drop task. (**b**–**f**) We successfully performed a modified bean drop task by using the real-time stitched video without any physical camera maneuver. The red dotted circles and arrows in each figure show initial and end positions and trajectories of the moving beans picked by a grasper. (**g**,**h**) The captured images from two single cameras show that a single camera cannot cover the saucer and the inverted cup simultaneously.

We also successfully performed a modified bean drop task in the simulator box by using the TCA (see Figure 8, Video S4). Figure 8a–f are captured images from the real-time stitched video. The first bean was grasped by a grasper (Figure 8b) and dropped into the hole of the inverted cup placed in the forward direction (Figure 8c). The other three beans were also grasped and dropped into the holes of the inverted cups placed in the left (Figure 8d), backward (Figure 8e), and right (Figure 8f) directions. In this modified bean drop task, as in the standard one, the operator always watched the real-time stitched video on the operating monitor during the task and used only one hand to perform the task without any physical camera maneuver. In contrast, the captured images from two single cameras (Figure 8g,h) show that a single camera cannot cover the saucer and the inverted cups simultaneously.

## 4. Discussion

We developed the TCA and real-time video stitching algorithm and demonstrated their high performance using laparoscopic surgical training tasks. For a large FoV, multiple videos from five different cameras were stitched by using our stitching algorithm and the latency of stitched videos was minimized by a multi-threading method. The TCA can provide larger FoV than commercial laparoscopic cameras. We performed a standard and modified bean drop task to verify the performance of the TCA and video stitching algorithm.

The TCA can provide a large FoV, eliminate the needs of physical maneuvering of the laparoscopic camera, and free up a surgical port. The large FoV of the operating scene allows the operator to easily manipulate a grasper in all directions, including a backward direction. In the current laparoscopic systems, which are based on one single camera, the visualization is best suited for the forward direction. Hence, the left, right and especially the backward bean drop, is challenging to carry out, using the current system. Even experienced surgeons, find it difficult when operating against or into the camera, in the current system. In addition, the operator could handle the modified bean drop task without any additional camera maneuvers, thanks to the larger FoV. The left, right, and backward bean drops were performed with as much ease as a forward bean drop. This advantage of large FoV visualization can potentially eliminate the need for a dedicated assistant operating the laparoscopic cameras, as is required in the current system. Moreover, the inner part of the surgical port is not occupied by the cameras, during an operation, as the multiple cameras are deployed and flared outside of the port. Therefore, the TCA can reduce the number of ports or free up a surgical port during an operation, which is a prominent advantage.

In this study, we used commercially available miniaturized cameras to make the TCA. The commercial cameras have their own design for the lens, cables, and image sensors. These design parameters of cameras affect the design of the TCA, especially for the port diameter. Because the design of the commercial cameras is not optimized for our specific application, the TCA in this study has a larger port diameter than the commercial ones. In future work, we will develop our own miniaturized cameras and thus decrease the port diameter to the same length as that of the commercial ones.

The TCA has four aluminum arms for deploying multiple cameras, which occupy some space depending upon the arm length. However, as the intra-abdominal space is limited during laparoscopic surgery, the arm length should be as short as possible. The current TCA has 5 cm arm lengths and the orientation of the cameras are all parallel. The arm length can be shortened by adjusting the camera angles. Further development is needed for optimizing the arm length and the camera angles.

We used an illumination system of the simulator box for imaging but the TCA does not have an illumination system. In this study, however, our first focus was to develop the TCA and the video stitching algorithm to demonstrate the advantages of the large-FoV visualization for laparoscopic surgery. In future work, we will add an illumination system into the TCA.

Image stitching, as performed in this study, relies on a couple of assumptions. In order to speed the algorithm up to work on real-time video, as is necessary for surgical use, we assumed that our cameras would remain stationary relative to each other. Thus, if the camera array is used in such a way that the array is bent or deformed in any way, this method will fail to produce a good result. The second assumption has to do with the projection model of image stitching. When a camera is moved translationally through space, it will witness parallax, the phenomenon by which closer objects seem to move much faster than farther away objects. Parallax can lead to artifacts appearing in the stitched image, such as those we can see in Figure 8, where the cups seem to be misaligned. Since the image stitching is performed without any knowledge of the 3D geometry of the scene, it relies on the assumption that the portion of the scene that needs to be stitched lies approximately on a single plane in 3D space. These artifacts will become more noticeable as the distance between the cameras increases, the scene becomes less planar, or the cameras move closer to the scene. While some methods have been proposed for correcting parallax discontinuity [28,29,30], none of them have yet proved reliable and fast enough to be used to completely correct for parallax in the surgical setting. We are working on a real-time 3D reconstruction that can potentially remove parallax artifacts.

In order to validate the potential clinical benefits of the large-FoV visualization, in the next phase, we will recruit surgeons, at various levels of training and experience, to perform numerous laparoscopic surgical tasks by using both current laparoscopic cameras and the TCA and make a comparison of the performance.

## 5. Conclusions

The TCA offers large-FoV imaging, improves the efficiency of operation, and enlarges operation range. Moreover, the TCA frees up a surgical port and potentially eliminates the need for physical maneuvering of the laparoscopic camera by the surgeon or an operative assistant. Further development of the TCA and video stitching algorithm can offer a high-performance, more efficient imaging system for laparoscopic surgery.

## Supplementary Materials

The following are available online at http://www.mdpi.com/2072-666X/9/9/431/s1, Video S1: The setup and performance of a modified bean drop task. Video S2: Standard bean drop task. Video S3: Standard bean drop task without the mark. Video S4: Modified bean drop task.
